# Robust Inference for Generalized Linear Mixed Models: A “Two-Stage Summary Statistics” Approach Based on Score Sign Flipping

**DOI:** 10.1017/psy.2024.22

**Published:** 2025-01-03

**Authors:** Angela Andreella, Jelle Goeman, Jesse Hemerik, Livio Finos

**Affiliations:** 1 University of Trento, Italy; 2 Leiden University Medical Center, The Netherlands; 3 Erasmus University Rotterdam, The Netherlands; 4 University of Padova, Italy

**Keywords:** generalized linear mixed model, longitudinal data, robustness, score test, sign flipping, two-stage summary statistics

## Abstract

Despite the versatility of generalized linear mixed models in handling complex experimental designs, they often suffer from misspecification and convergence problems. This makes inference on the values of coefficients problematic. In addition, the researcher’s choice of random and fixed effects directly affects statistical inference correctness. To address these challenges, we propose a robust extension of the “two-stage summary statistics” approach using sign-flipping transformations of the score statistic in the second stage. Our approach efficiently handles within-variance structure and heteroscedasticity, ensuring accurate regression coefficient testing for 2-level hierarchical data structures. The approach is illustrated by analyzing the reduction of health issues over time for newly adopted children. The model is characterized by a binomial response with unbalanced frequencies and several categorical and continuous predictors. The proposed approach efficiently deals with critical problems related to longitudinal nonlinear models, surpassing common statistical approaches such as generalized estimating equations and generalized linear mixed models.

## 1 Introduction

Generalized linear mixed models (GLMM) play a pivotal role in psychometric data analysis due to their ability to handle clustered data structures such as longitudinal data when the response variable follows a non-normal distribution, e.g., repeated measures of a binary item across subjects (Cnaan et al., 1997; Tuerlinckx et al., 2006). Despite their popularity, important drawbacks are sensitivity to model violations and computational issues. Flexibility in dealing with a complex data structure is coupled with a strong reliance on the underlying parametric assumptions. Additionally, this flexibility poses challenges for researchers in specifying the fixed and random components within the model; this sensitive issue has generated a lively discussion within the scientific community (see, for examples Barr et al., 2013; Bates, Kliegl, et al., 2015; Matuschek et al., 2017).

As a prototypical example, in this paper, we make use of the dataset on *follow-up* reports of Italian children adopted from foreign countries presented in Santona et al. (2022). The dataset comprises 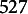 children. The follow-up questionnaires are administered to children at different times (depending on the regulations of the foreign countries) and range from 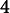 to 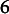 follow-ups, for a total of 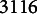 observations. The health status is measured through the analysis of several questions. The research aim is to explore the adaptation of adopted children to their new family and the social environment during the early stages of adoption, as well as how this adaptation evolves over time. In this analysis, we will focus on the presence or absence of health issues (i.e., a binary variable). The within-subject variable is then the month from the adoption, while other subject-specific socio-demographic variables (i.e., gender, age, country of origin) are also included in the model.

The fitting of a GLMM with a logistic link function and a binomial response reveals initial difficulties. One of them is that the default optimization method fails to converge, leading to unreliable model parameter estimates. Using alternative constrained optimization methods based on quadratic approximation is necessary by setting a high threshold for the number of times the function can be evaluated within the algorithm, which leads to a large increase in computational time and space complexity. In addition, as mentioned before, the specification of the GLMM is not so straightforward. The researcher must decide which variables are considered as random effects and which type of random effect must be inserted into the model. These decisions directly impact the correctness of statistical inference on the coefficients, and the proper choice between a parsimonious model (Bates, Kliegl, et al., 2015; Matuschek et al., 2017) and a maximal model (Barr et al., 2013) is still an open problem.

In order to deal with these problems, this paper presents a robust approach for coping with inference on binary responses with a complex correlation structure, e.g., longitudinal data. The method can deal with any dependent variable following a generalized linear model (GLM) (Agresti, 2015) under the usual regularity condition (Azzalini, 2017). As pointed out at the beginning of the introduction, problems related to the popular generalized linear mixed models are numerous and are summarized below.

First, generalized linear (mixed) models are frequently misspecified due to overdispersion, heteroscedasticity, or neglecting nuisance variables. In case of misspecification, traditional parametric tests (i.e., tests that exclusively depend on an assumed parametric model to compute the null distribution of the test statistic) may lose their validity as they rely on estimating the Fisher information under incorrect assumptions (Heagerty & Kurland, 2001). An alternative approach for testing regression coefficients in potentially misspecified GLMs is to employ a Wald-type test with a robust variance estimator (i.e., the sandwich estimator). However, for small to moderate sample sizes, sandwich estimates are rather inaccurate, leading to overly liberal tests (Hemerik et al., 2020). Moreover, existing quasi-likelihood methods (e.g., Generalized Estimating Equation (GEE), which can be defined as a generalization of the GLM to longitudinal data (Carlin et al., 1999; Diggle et al., 1994; Laird & Ware, 1982; Liang & Zeger, 1986)) for testing in misspecified models often fail to provide satisfactory control over the type I error rate as underlined by Hemerik et al. (2020). Even when well-specified, these tests control type I errors only asymptotically. In particular, GEE handles the dependence between observations by specifying a working correlation matrix. The choice of this matrix significantly affects regression estimator efficiency (Crowder, 1995; Sutradhar & Das, 2000; Wang & Carey, 2003). The working correlation is barely known in practice, and choosing the identity (i.e., assuming independence between the repeated measurements) is a common choice to have consistent estimators. GEE fails to control type I error in small samples, unbalanced designs, endogenous covariates when assuming independence, and not randomly missing values (Hemerik et al., 2020; Vonesh & Chinchilli, 1996; Wang & Carey, 2003).

Second, the estimation process of generalized linear mixed models, having no closed-form solution for the likelihood function, can encounter convergence issues, especially in the presence of complex random effect structures. The main factors contributing to these problems include high dimensionality, non-identifiability, singular design matrices, imbalanced and sparse data, and high sensitivity to starting values, even if strong assumptions are made to simplify the estimation process. Again, these lead to invalid inferences, increased computational time, and resource use.

In summary, model misspecification and convergence issues are important concerns that can impact the reliability of inferences. The robustness of the significance tests depends not only on the degree of agreement between the specified mathematical model and the actual population data structure but also on the precision and robustness of the computational criteria for fitting the specified covariance structure to the data.

To address these challenges, nonparametric methods, such as permutation tests, prove useful for testing the effects of covariates. It is well known that permutation theory has emerged as a promising alternative to traditional, parametric statistical approaches, providing more robust and reliable inference for various hypothesis testing scenarios (Pesarin, 2001). Kherad-Pajouh and Renaud (2010) and Lee and Braun (2012) proposed nonparametric tests for linear mixed models. The first is based on permuted residuals, while the second is based on the best linear unbiased predictors and restricted likelihood ratio test statistic. However, both rely on the Gaussian assumption distribution of the outcome. Instead, Basso and Finos (2012) and Finos and Basso (2014) proposed a permutation test for the generalized linear mixed model based on the parametric statistical test. Basso and Finos (2012) and Finos and Basso (2014) fit a model for each cluster/subject, and then the parameter of interest is estimated and tested considering the within-subject estimated coefficients as responses. Basso and Finos (2012) deal with testing within-subject effect while Finos and Basso (2014) the between-subject one. The test proposed by Basso and Finos (2012) is exact but has low power since the power of the statistical test depends on the quality of estimating the covariance of the cluster-level parameter estimator. In contrast, the statistical test presented by Finos and Basso (2014) is exact only in the case of balanced number of within-subject observations. In addition, the approach of Finos and Basso (2014) heavily relies on an adequate estimate of the Fisher Information.

This paper investigates challenges and novel methodologies for testing regression coefficients in generalized linear mixed models. We rely on the resampling-based procedure recently proposed by Hemerik et al. (2020) and De Santis et al. (2024) to solve the above-mentioned problems. We extend their approach, dealing with the within-subject dependence that characterizes longitudinal data and the heteroscedasticity between groups of observations. The test proposed by Hemerik et al. (2020) computes *effective scores* and randomly sign-flips each subject’s contribution to the score. The method is improved in De Santis et al. (2024) by including an additional standardization step. This leads to a test that has excellent small-sample performance and is asymptotically valid under any variance misspecification. The method proposed here exploits the “two-stage summary statistics” concept, which is well-known in several applied fields such as neuroimaging (Helwig, 2019; Mumford & Poldrack, 2007) and clinical trials (Everitt, 1995; Frison & Pocock, 1992). The works of Basso and Finos (2012) and Finos and Basso (2014) mentioned before also involve using summary measures at the subject/cluster level. However, these two methods, as well as other permutation-based statistical tests (i.e., Kherad-Pajouh and Renaud, 2010; Lee and Braun, 2012), cannot deal with heteroscedasticity and unbalanced data. In addition, most of these methods depend on some parameter estimation (e.g., Fisher Information in Finos and Basso, 2014) or specification of the random part of the model. In contrast, the approach proposed is free from assumptions concerning the random data structure.

The article is organized as follows. Section 2 outlines the sign-flipping test proposed by De Santis et al. (2024). This approach is extended to clustered data in Section 3. Section 4 evaluates by simulations the performance of our method in comparison with the GLM, GLMM, and GEE testing in terms of type I error control and power. Finally, Section 5 presents our case study, highlighting how, thanks to the proposed approach, it is possible to provide robust inference for binary longitudinal data.

The method proposed is efficiently implemented in the R package jointest (Finos & Andreella, 2024), which is compatible with large datasets and complex statistical models. Along the manuscript, we will call the approach developed by Hemerik et al. (2020) and improved by De Santis et al. (2024) the *flipscores* approach, while the proposed extension to deal with dependent observations as *flip2sss* (i.e., flipscores two-stage summary statistics). We also clarify here that when we use the term *score*, we refer to the score function derived from the likelihood of GLMs where only the fixed components are assumed. This should not be confused with scores obtained from item theory models.

## 2 Sign-flipping score test with independent observations

We introduce the *flipscores* method proposed by De Santis et al. (2024) for inference on regression coefficients in GLM, which improves Hemerik et al. (2020)’s approach, particularly in the case of small sample size. The statistical test is based on randomly flipping the signs of scores (i.e., one score for each subject), which is robust to misspecification of the variance in the GLM framework. The notation used throughout the manuscript follows that used by De Santis et al. (2024).

Consider *n* independent observations 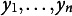 following a GLM from the exponential dispersion family (Agresti, 2015): 

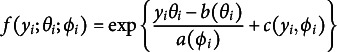

where 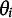 is the canonical parameter, 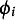 the dispersion one, and 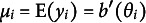 . The generalized linear model is then defined as: 

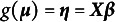

where 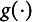 is the link function, 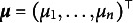 , 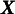 is the design matrices corresponding to the vector of *q* parameters 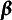 .

The main focus is to test the null hypothesis for a given element *d* of 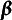 still accounting for all nuisances parameters, i.e., 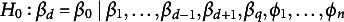 . The case of one parameter of interest, i.e., one column of matrix 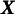 , is then considered. However, the approach is extendable to the multivariate case (De Santis et al., 2024; Hemerik et al., 2020). The test statistic used in Hemerik et al. (2020) (with a notation slightly adapted to our context) is the *effective score*: 
(1)

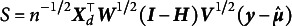

where 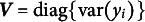 , 
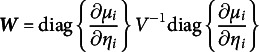
 , 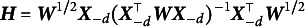 and 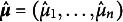 are the fitted values of the model under the null hypothesis. 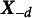 indicates the matrix 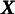 without the column *d* and 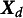 the one containing only the column *d*. Since *S* is a score test, 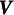 and 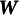 are computed under the null hypothesis (i.e., assuming 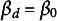 ), furthermore being 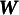 a diagonal matrix, its square root is the element-wise square root.

To improve the small-sample reliability of the test in Hemerik et al. (2020), De Santis et al. (2024) proposed the standardized version of (1), i.e., 
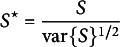
 where 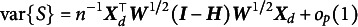 . 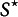 results to be asymptotically valid under any variance misspecification. It is important to remark that the tests by Hemerik et al. (2020) and De Santis et al. (2024) are asymptotically equivalent. In particular, they share the same robustness to variance misspecification. The test by De Santis et al. (2024) is, however, better for small sample size since it has better “level accuracy” (i.e., the level of the test tends to be closer to 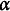 ).

The statistic *S* in Equation (1) can be rephrased as a sum of *n* components, i.e., the effective score contributions. The associated *p*-value is computed by randomly flipping the signs of these score contributions. We write each sign-flipping transformation as an 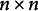 diagonal matrix 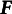 , with diagonal elements that are 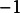 or 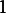 with equal probability. Sign-flipping the score contribution means multiplying the effective score by a given sign-flip diagonal matrix 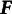 , i.e.: 
(2)

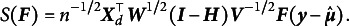



The corresponding standardized version (De Santis et al., 2024) equals 
(3)

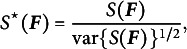

where 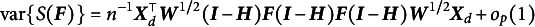 .

Considering *B* independent sign flip transformations, where 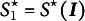 is the observed test statistic, we reject the null hypothesis 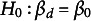 versus the alternative 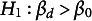 at significance level 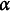 if: 

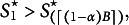

where 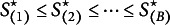 are the sorted statistics and 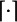 is the ceiling function. In the same way, we reject 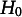 versus 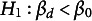 if 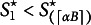 and versus 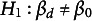 if 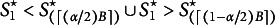 .

As said above, the method is easily extendable to nonscalar *d* (De Santis et al., 2024; Hemerik et al., 2020). In short, 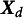 becomes a matrix instead of a vector, the *effective score* test statistic 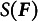 defined in Equation (2) becomes a vector, and the 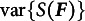 in Equation (3) is now a matrix. The test statistic for 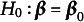 , with 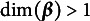 , equals: 
(4)

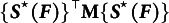

where 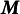 is generally defined as the inverse of an estimate of the effective Fisher information of 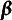 , i.e., Mahalanobis distance (Pesarin, 2001). Note that we do not need to estimate the Fisher Information correctly due to the robustness of our method against variance misspecification.

Having, therefore, the null distribution of the score-based test statistic 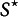 , the following section shows how to deal with the case of correlated longitudinal data. The proposed solution will heavily rely on the robustness of the *standardized flipscores* to variance misspecification since this will allow us to avoid modeling the random part of the mixed model.

## 3 Extension to the non-independent case

This section extends the test from Section 2 to the case where the dependent variable has a clustered structure, e.g., longitudinal data. To make the proposed solution more concrete, we briefly introduce the part of the data that will be analyzed in Section 5. Adopted children are measured longitudinally over Time, i.e., in different time points and different number of occasions. Among others, health issues are recorded; this is the response variable Unhealth (binary variable). Other variables in the model, such as Sex (binary variable), Age (of the child arrived in the family, in years), and Country (of origin), are constant within the subjects and play the role of between-cluster variables. A possible model of interest is the following: Unhealth 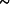 1+ Country + Age + Sex + Time + Sex:Time.

Consider *n* observations 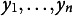 with a correlation structure dictated by the longitudinal data nature. We have then 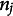 observations in cluster (i.e., child) *j* with 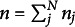 and *N* is the total number of clusters (i.e., children). We then assume that the observations within the clusters are dependent (e.g., repeated measurements for each subject). The exchangeability assumption used to compute the null distribution of the standardized score test statistic 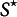 defined in Equation (3) does not hold.

The extension to the non-independent observations case proposed is based on the well-known “two-stage summary statistics” approach (Everitt, 1995; Frison & Pocock, 1992; Helwig, 2019; Mumford & Poldrack, 2007). In short, the “two-stage summary statistics” approach reduces the hierarchical complexity of data as longitudinal ones by computing summary measures at the cluster level in the first stage, which are then analyzed as a response random variable in the second stage. Therefore, the main assumption is to have (at least asymptotically) unbiased estimators of these summary measures.

In the first stage, a generalized linear model can be fitted separately for each subject 
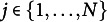
 including only the *h* covariates 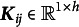 with 
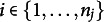
 that vary within-cluster *j*, i.e., 
(5)

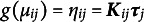

where 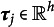 the vector of *h* parameters of the cluster *j*, 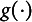 is the link function and 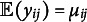 with 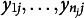 following a distribution from the exponential dispersion family (Agresti, 2015) 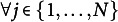 . Fitting the model defined in Equation (5) leads to a vector of estimated parameters 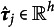 for each cluster *j* corresponding to the design matrix 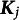 that vary within-subject *j*. Regarding our example, a natural choice would be to model each subject with logistic regression by Unhealth 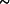 1 + Time, that is, 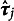 would be the vector containing the estimated intercept and slope for each child *j*, i.e., our “summary statistics”.

In the second stage, the *N* vectors 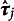 of estimated coefficients of each cluster *j* is collected in a 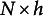 matrix 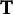 and modeled by a linear model having the between-cluster variables as predictors: 
(6)

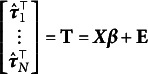

where 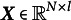 is the matrix of between-cluster variables (i.e., Intercept, Country, Age and Sex in our example), 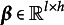 is the matrix of predictors where each column relates to a different column of 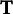 , and 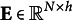 is the matrix of errors.

Consider again the model of interest presented in Section 5, i.e., Unhealth 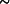 1+ Country + Age + Sex + Time + Sex:Time. The intercept of within-cluster effects (i.e., the first column of 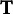 ) is modeled by the between-cluster effects Intercept, Country, Age and Sex. Instead, the estimated slope related to Time (i.e., the second column of 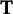 ) is modeled only by Intercept and Sex covariates. Therefore, in the second column of 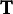 , only those coefficients of Intercept and Sex covariates will be estimated, while the ones associated with Country and Age are set to 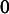 .

In summary, the problem is now rewritten as a multiple multivariate linear model where each observation is 
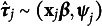
 with 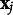 is the *j* row of 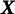 from Equation (6). The variance of the error terms 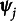 is the variance of the independent rows of the error matrix 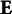 defined in Equation (6), i.e., 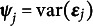 where 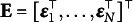 . In particular, 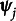 can be decomposed by two sources of variability: the variance of the random effects (named 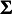 ) and the conditional variance of 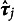 (named 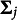 ), i.e., 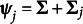 . The first captures the between-subject variability due to the presence of random effects, while the second represents the within-cluster variability.

Outside the fully balanced designs with homoscedastic errors, the 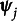 varies among clusters, making the standard linear model tools unreliable. The literature on “two-stage summary statistics” focuses on the estimate of these quantities (Beckmann et al., 2003; Finos & Basso, 2014; Frison & Pocock, 1992; Helwig, 2019; Mumford & Poldrack, 2007) hence making assumptions on the data and being constrained to a precise specification of the random quantities in the model. In our data example, the researcher employing standard “two-stage summary statistics” approaches should choose whether intercept and slope are random coefficients and also among independent or correlated random effects–decisions that are not required with the approach we propose here.

The robustness of the *standardized flipscores* approach to variance misspecification results in an ideal tool to make inference on the models used in the second stage defined in Equation (6). The null hypothesis 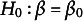 is then tested with the standardized score test defined in Equation (4), applying the same sign-flipping transformation across all *h* models, still avoiding the complexities associated with formulating the random component of the model. In fact, the debate about how to formulate the random part inside the GLMM framework, i.e., choosing between a parsimonious (Bates, Kliegl, et al., 2015; Matuschek et al., 2017) or “maximal” (Barr et al., 2013) model, is open.

A further remark relates to fitting separated models for each cluster. Actually, any summary measures at the cluster level can be used in the second stage; the only required property is the (asymptotic) unbiasedness of these summary measures. As an example, in the application of Section 5 and in the simulations of Section 4, we will adopt and evaluate the use of the Firth correction (Firth, 1995) in the fitting of the cluster-level binomial models, i.e., Equation (5); this helps in reducing the finite-sample bias of the maximum likelihood estimates. Although the unbiasedness property holds, the selection of summary measures is guided by the researcher’s specific objectives. For instance, in clinical trials, common choices include post-treatment means or mean changes relative to baseline (Frison & Pocock, 1992). In our example, we focus on the effect of time on health conditions.

## 4 Simulations

This section explores the proposed approach and compares it with the GLM, GLMM, and GEE. The comparison is in terms of type I error control and power. The dependent variable 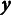 is simulated as a Bernoulli random variable with the following mean: 
(7)

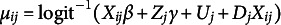

where 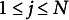 refers to cluster *j*, which contains 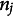 observations, so that 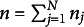 . The 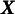 and 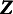 matrices are the design matrices of the tested and nuisance parameters 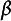 and 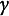 . 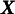 and 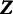 are correlated and simulated as 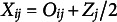 where 
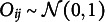
 and 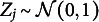 . Finally, the matrix 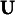 defines the subject-specific random effect (i.e., random intercept) and 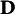 the subject-specific random slope of 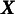 .

Since the proposed approach can deal with different types of correlation structures, in these simulations, we consider the case of full random effects (i.e., 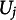 and 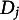 present). We decided to follow Equation (7) to have simulations reflecting the scenario of the follow-up application proposed in Section 5. The ones considering a subject-specific nuisance covariate with and without the presence of an additional random effect for 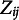 are placed in Appendix A. The aim is to test 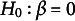 under this correlation structure. We take 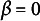 to evaluate type I error control and 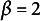 to analyze the power of the approaches. The nuisance parameter is set to 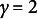 . The subject-specific random effects 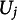 and 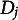 are simulated from an uncorrelated bivariate normal distribution with mean 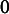 and standard deviation 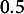 . In both scenarios, 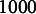 simulations are performed.

The *flip2sss* approach is proposed in two versions. The first one (called in the simulations’ results simply “flip2sss”) uses the maximum likelihood estimator of the classical generalized linear model in the first stage to estimate the summary statistics. The second one (called in the simulations’ results “flip2sss Firth”) utilized instead the modified score equations proposed by Firth (1995) applied to the logistic model (Heinze & Schemper, 2002; Kosmidis & Firth, 2009; Puhr et al., 2017) in the first stage. In fact, the main assumption of the method proposed is having unbiased (at least asymptotically) estimates of the parameter at the first stage defined in Equation (5). The bias reduction approach proposed, i.e., the Firth correction, permits dealing with the problem of “perfect separation” (Albert & Anderson, 1984) also called “monotone likelihood” (Bryson & Johnson, 1981) when we estimate the summary statistics in the first stage. However, several bias reduction techniques are present in the literature (e.g., Cordeiro and McCullagh, 1991; Kenne Pagui et al., 2017; Kosmidis and Firth, 2021) that can be used instead of the Firth correction.

We then fit two GLMMs: The first one is the correctly specified (called in the simulations’ results simply “GLMM”), i.e., considering both a random intercept and a random slope in the model specification since 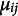 depends on 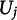 and 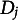 in Equation (7). The second one is misspecified (called in the simulations’ results “GLMM misspecified”), i.e., we define only the random intercept in the model specification while 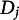 in Equation (7) is simulated.

The GEE fitted here refers to assuming the independent working correlation matrix. This choice is justified since the consistency of GEEs is not assured in the case of using an arbitrary working correlation matrix when the covariates 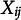 can vary within a subject over time (Sullivan Pepe & Anderson, 1994). The independent working correlation matrix is typically a safe choice even though the independence assumption is not necessarily valid.

Although these simulation analyses compare the performance of different models in terms of type I error control and power, we must remark that the models defined above have distinct inferential objectives. Specifically, the GLMM provides conditional estimates, while the GEE yields marginal ones. Consequently, the interpretation of the estimated parameters is not directly comparable (Gardiner et al., 2009).

In addition to the GEE and the two GLMMs described above, we also fit a standard GLM with a logit link function.

**Figure 1:**
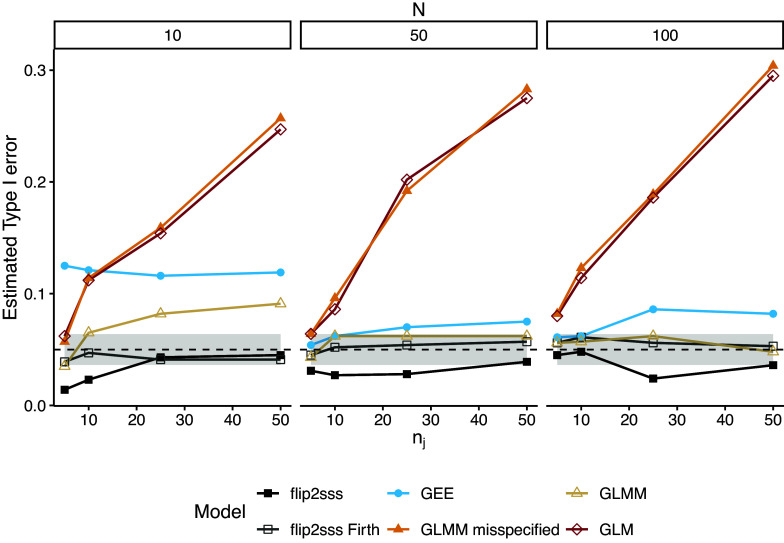
Estimated type I error considering 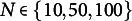 number of subjects and 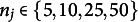 repeated measurements. Each line represents one model, and the grey area around the dashed black line represents the 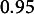 confidence bound for 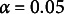 . The term “GLMM” refers to the GLMM with random intercept and random slope specified, whereas “misspecified GLMM” refers to the GLMM with solely the random intercept included in the model specification. The term “flip2sss” refers to the results using the maximum likelihood estimator in the first stage, while the term “flip2sss Firth” refers to the results using the bias correction proposed by Firth (1995). Finally, the terms GLM and GEE refer to a GLM with a logit link function and a GEE with an independent working correlation matrix, respectively.

**Figure 2:**
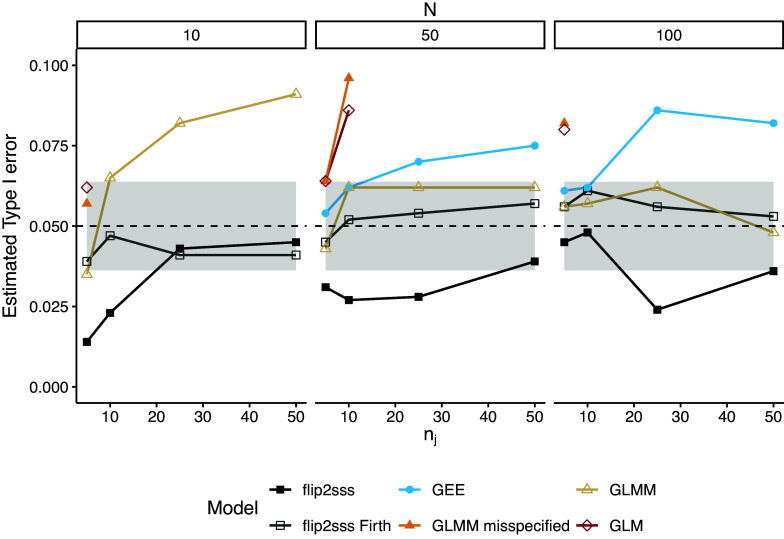
Zoom of Figure 1 considering the estimated type I error 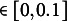 . Estimated type I error considering 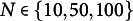 number of subjects and 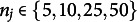 repeated measurements. Each line represents one model, and the grey area around the dashed black line represents the 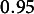 confidence bound for 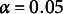 . The term “GLMM” refers to the GLMM with random intercept and random slope specified, whereas “misspecified GLMM” refers to the GLMM with solely the random intercept included in the model specification. The term “flip2sss” refers to the results using the maximum likelihood estimator in the first stage, while the term “flip2sss Firth” refers to the results using the bias correction proposed by Firth (1995). Finally, the terms GLM and GEE refer to a GLM with a logit link function and a GEE with an independent working correlation matrix, respectively.

Figure 1 shows the estimated error rate considering 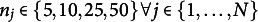 , and 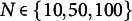 , while Figure 2 presents only values of the estimated error between 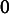 and 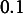 to have a clearer vision about the difference on controlling type I error between the methods. First, we can note that the misspecified GLMM and the basic GLM do not control the type I error for small to large sample sizes. The correctly specified GLMM controls the type I error in the case of 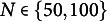 , while GEE fails in most of the scenarios considered. In contrast, the proposed *flip2sss* approach with Firth correction maintains type I error control in all scenarios, while we are conservative if the Firth correction is not used in the first stage. Across all the simulations, the correctly specified GLMM encounters a convergence problem 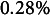 of the time and a singularity issue 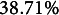 of the time.

**Figure 3:**
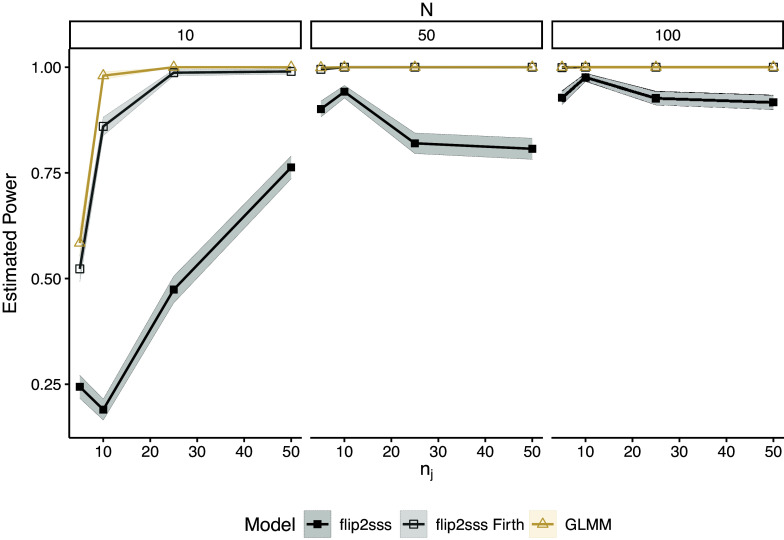
Estimated power considering 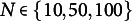 number of subjects and 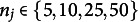 repeated measurements. Each line represents one model with corresponding colored confidence bounds at 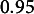 level. The term “flip2sss” refers to the results using the maximum likelihood estimator in the first stage, while the term “flip2sss Firth” refers to the results using the bias correction proposed by Firth (1995).

Figure 3 shows the estimated power under the same settings of Figure 1 for 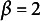 . In this case, we only show the results for the two versions of *flip2sss* and correctly specified GLMM since the GLM, GEE, and misspecified GLMM did not control the type I error in any scenario. We see that the *flip2sss* with Firth correction maintains high power as the correctly specified GLMM in most of the scenarios.

On average, full separation occurs 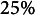 of the time when 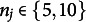 and 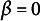 . In contrast, full separation occurs 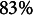 of the time for 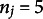 and 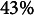 of the time for 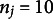 when 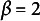 . These percentages decrease as 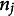 increases. In addition, on average, we observe only 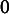 s in 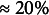 of cases when 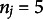 and 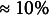 of cases when 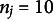 regardless of whether 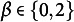 .

**Figure 4:**
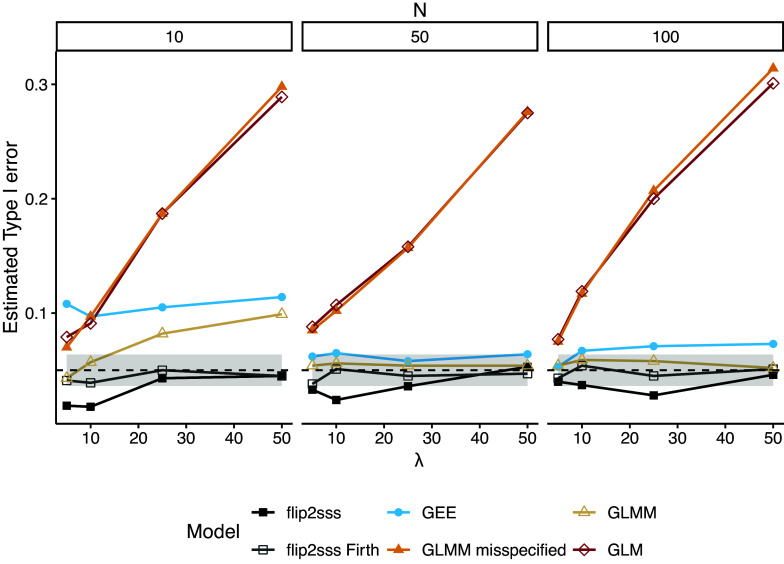
Estimated type I error considering 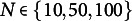 number of subjects and 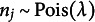 where 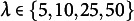 repeated measurements, i.e., in the case of unbalanced data. Each line represents one model, and the grey area around the dashed black line represents the 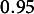 confidence bound for 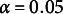 . The term “GLMM” refers to the GLMM with random intercept and random slope specified, whereas “misspecified GLMM” refers to the GLMM with solely the random intercept included in the model specification. The term “flip2sss” refers to the results using the classic GLM in the first stage, while the term “flip2sss Firth” refers to the results using the bias correction proposed by Firth (1995). Finally, the terms GLM and GEE refer to a GLM with a logit link function and a GEE with an independent working correlation matrix, respectively.

Figure 4 shows the estimated type I error in the case of unbalanced data. Data are simulated following again Equation (7) with 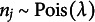 where 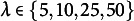 . We note the ability of the *flip2sss* approach proposed to handle unbalanced data in contrast to GEE. The method proposed is conservative in the case of a small number of repeated measurements if we do not use the Firth correction in the first stage. At the same time, the performance of GLMM strongly depends on correctly specifying the random part of the model, and the type I error is not controlled in the case of a small number of subjects. When the correctly specified GLMM is fitted, in 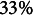 of the simulations, a singularity problem is present, while 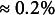 of the times is a convergence or identifiability issue.

**Figure 5:**
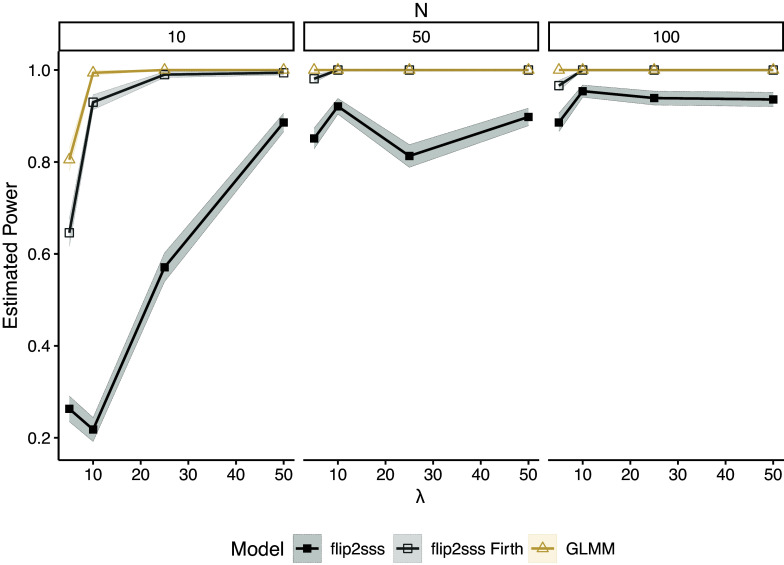
Estimated power considering 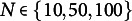 number of subjects and 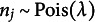 where 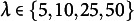 repeated measurements, i.e., in the case of unbalanced data. Each line represents one model with corresponding colored confidence bounds at 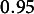 level. The term “flip2sss” refers to the results using the classic GLM in the first stage, while the term “flip2sss Firth” refers to the results using the bias correction proposed by Firth (1995).

Finally, Figure 5 shows the estimated power under the same settings of Figure 4 fixing 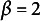 . We can note the low power of *flip2sss* approach without the Firth correction in the case of small sample sizes. However, using the Firth correction, the estimated power reaches the level of the correctly specified GLMM, controlling the type I error at the same time. The results for GEE, as well as the ones for GLM and the misspecified GLMM, are not reported since it does not control the type I error in any scenarios of Figure 4.

On average, complete separation occurs 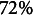 of the time for 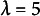 and 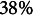 for 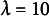 when 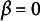 . If 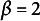 , 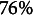 of the cases encounter a complete separation problem when 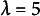 , while 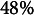 if 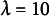 . In addition, we observe only 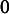 s approximately 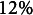 ( 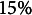 ) of the times on average when 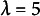 and 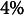 ( 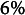 ) of the time when 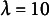 and 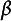 equals 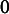 ( 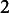 ).

We can conclude from these simulations that the proposed method controlled the type I error for both small and large samples and for both few and many repeated measurements in the case of balanced and unbalanced data, unlike the two competitive methods considered, namely GEE and GLMM. In addition, a downside of the GLMM is that it requires the user to correctly specify the mixed model, which is often a problematic task, as mentioned above (Barr et al., 2013; Bates, Kliegl, et al., 2015; Matuschek et al., 2017).

## 5 Application

Adoption is a legal process in which an individual or a couple assumes permanent parental responsibility for a child who is not biologically related to them. The complexity of the adoption journey varies depending on factors such as the child’s age, the circumstances surrounding the adoption, and the child’s prior experiences. The dataset analyzed comes from a study of Santona et al. (2022), which is based on responses given by adoptive parents to reports called *follow-ups*. Follow-ups are periodic reports that provide information on the progress of the adoption, the child’s adjustment to the new environment, and their psychological and physical well-being. The Authorized Entity (i.e., an Italian institution that manages the adoption procedures), following the return of the adoptive family to Italy with the child, must carry out mandatory follow-ups required of adoptive families by the country of origin, which must then be transmitted to the foreign authority.

The sample analyzed consists of 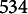 minors adopted between 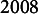 and 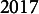 through the Italian Childhood Aid Center (CIAI). The minors range in age from 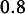 to 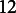 years upon arrival in Italy and come from different countries such as India, China, Burkina Faso, Colombia, Ethiopia, Thailand, Vietnam, and Cambodia. To study the trend over time of children’s physical development and health status detected at each follow-up, a new variable called Unhealth was created based on the following answers:

1. Physical development (regular versus irregular)
2. Psychomotor development (regular versus irregular)
3. Sleep-wake rhythm (regular versus irregular)
4. Nutrition (regular versus irregular)
5. Diseases (pathologies and/or developmental alterations versus none)

which belong to the life and medical areas of the follow-up report. The new variable, Unhealth, is defined as a binary variable where 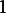 means that at least one of the above health problems is present and 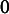 means none are present. In addition to the questions described above, we have various socio-demographic information for each child described in Table 1.

**Table 1:** Description of the socio-demographic variables.

**Variable name**	**Description**
Sex	Sex of the child (Male/Female)
Time	Discrete variable indicating the survey time
Age	Age of the child when they arrived in the family
Country	Country of provenience of the child
Subject	Discrete variable indicating the child

**Figure 6:**
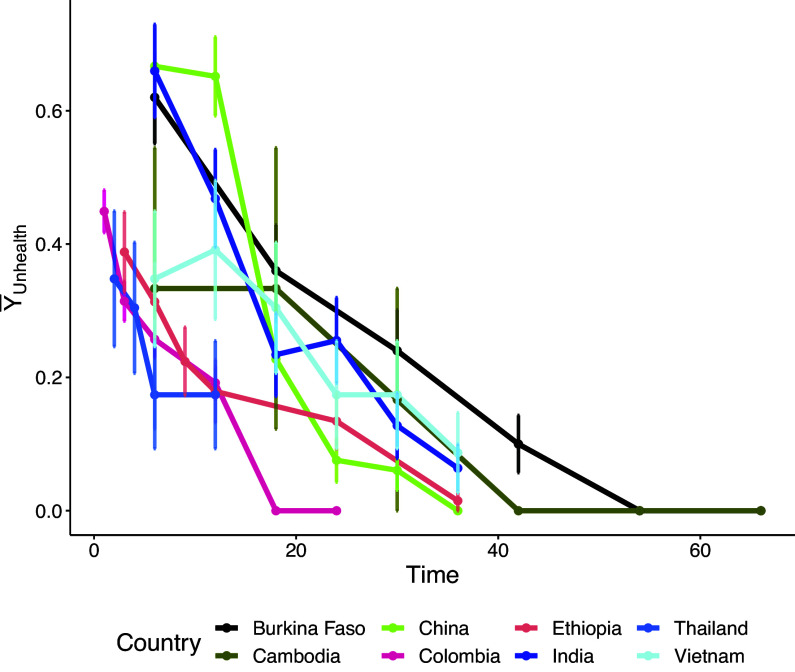
Sample proportion of children with an unhealthy status across time for each country. The colored bars indicate a 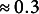 confidence interval for each time point/country combination. If bars are absent, it indicates that the variance of Unhealth equals 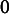 .

The health status is recorded multiple times for each child; the aim is to understand the development of this variable across time, taking into account the evident within-child variability. Covariates such as Age (median 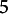 year, range: between 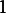 month and 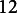 years), Country ( 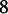 countries, proportions ranging from 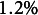 to 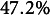 ), and Sex (female 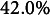 ) defined in Table 1 are considered in the model as moderator variables. Figure 6 shows the relation between country and time. As is visible from the plot, the number of follow-ups and the time from the adoption vary among countries. This is due to differing regulations.

Here, we apply our proposed method presented in Section 3 and two other competitive approaches common in the literature, i.e., GEE and GLMM. The packages jointest (Finos & Andreella, 2024), geepack (Højsgaard et al., 2006), and lme4 (Bates, Mächler, et al., 2015) are used respectively. We utilize the car package (Fox & Weisberg, 2019) to compute the ANOVA test for the generalized linear mixed models. The R command used for performing the analysis is described in Code 1 for the proposed approach and Code 2 in Appendix B for the GEE and GLMM methods.

**Figure:**
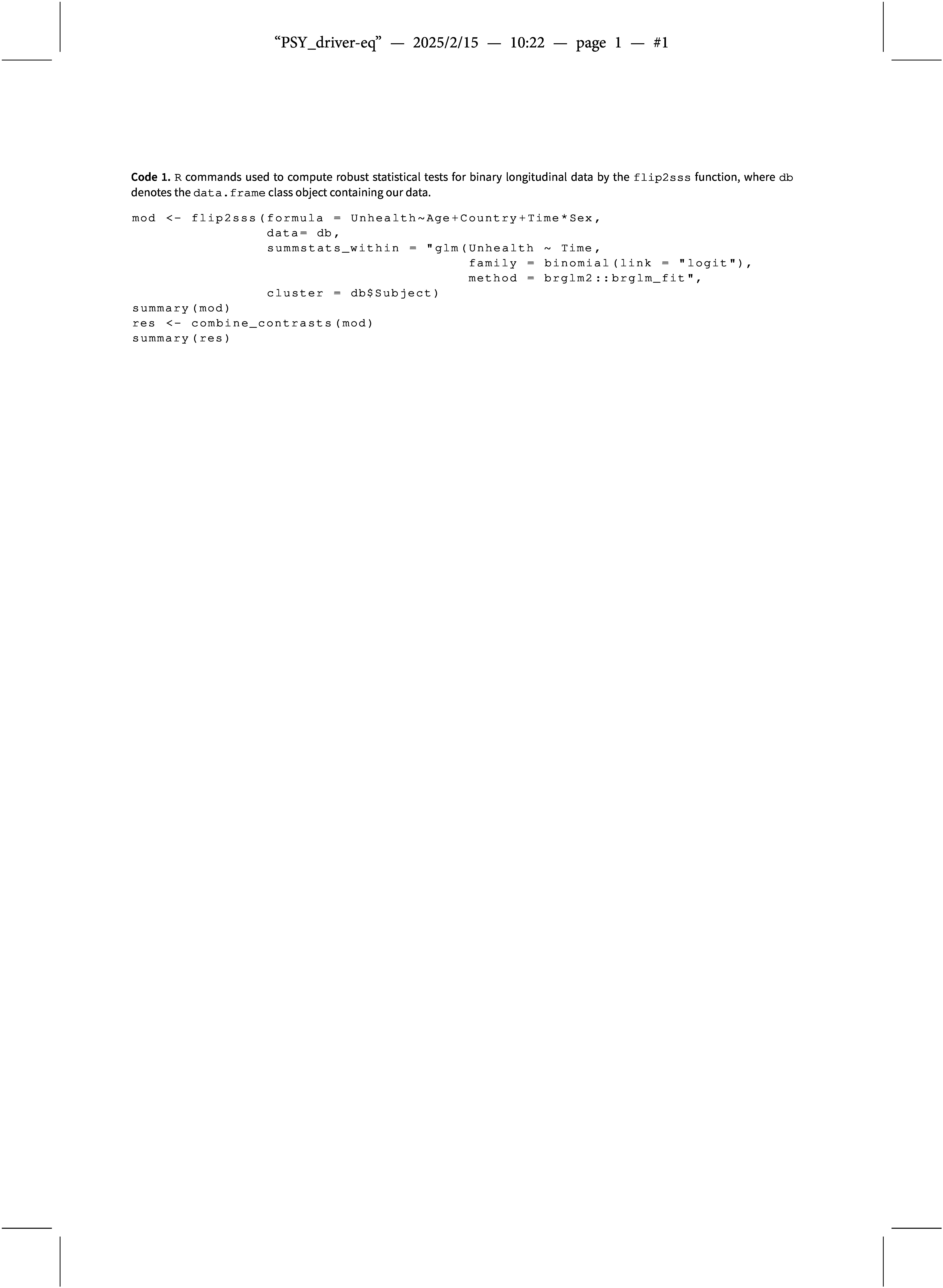

The random effects are directly managed by specifying the variable that identifies the cluster (e.g., subject) in the cluster and the subject-specific variable in the summstats_within arguments of the flip2sss function. Therefore, as mentioned in Section 3, the problem of defining the correlation structure related to the GEE and GLMM models is solved in a simple way. Specifically, we use the Firth correction (Firth, 1995) based on the brglm2 R package (Kosmidis, 2023) for fitting the model in the first stage specified in the summstats_within argument. The combine_contrasts function computes the global test for each covariate at the subject level defined in the formula argument of the flip2sss function, i.e., Age, Country and Sex having as dependent variable the estimated intercept and slope specified in the summstats_within argument of the flip2sss function. The Mahalanobis distance (used by default) is used as the combining function (Pesarin, 2001) specified in the comb_func argument of the combine_contrasts function and defined in Equation (4).

We start our analysis by estimating the GLMM with two random effects: a random slope defined by the survey time and a random intercept defined by the subject variable. The fixed effects part is specified in the same way as defined in the right part of the formula object in Code 1. Using the default optimizer, i.e., a two-stage optimization, where the Bound Optimization BY Quadratic Approximation (BOBYQA) iterative algorithm (Powell et al., 2009) is used in the first stage and the Nelder-Mead approach (Lagarias et al., 1998) in the second one, the estimation process does not converge. This was solved by specifying the BOBYQA optimizer for both stages, increasing the maximum number of function evaluations to 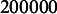 , leading to a high computational time (i.e., 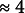 minutes). In contrast, GEE and flip2sss reach a computational time equal to 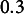 and 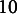 seconds, respectively.

Table 2 shows the results of the *flip2sss* analysis. The ANOVA-like test performed by the combine_contrasts function detects significant effects for all variables included in the model. More specifically, the time from the arrival in the new family and the age of arrival decrease the probability of health issues, while males are more prone to them.

**Table 2:** *flip2sss* model results: summary of the model and related Anova-like table.

**Model**	**Coefficient**	**Estimate**	**Score**	**Std. Error**	*z* **value**	**Part. Cor**	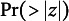
.Intercept.	Intercept	0.7134	13.1132	6.7978	1.9290	0.1407	0.1000
.Intercept.	Sex Male	0.9456	43.2835	11.0794	3.9066	0.2849	0.0002
.Intercept.	Country Cambodia	−1.0258	−2.7490	2.5775	−1.0666	−0.0778	0.2953
.Intercept.	Country China	0.8602	12.6129	6.0806	2.0743	0.1513	0.0930
.Intercept.	Country Colombia	−0.6174	−11.7067	6.8882	−1.6995	−0.1240	0.1428
.Intercept.	Country Ethiopia	−1.1686	−15.8878	5.9028	−2.6916	−0.1963	0.0148
.Intercept.	Country India	0.4292	5.6177	5.6934	0.9867	0.0720	0.4255
.Intercept.	Country Thailand	−1.2504	−9.9562	4.4885	−2.2182	−0.1618	0.1020
.Intercept.	Country Vietnam	−0.9933	−10.0863	5.0558	−1.9950	−0.1455	0.0558
.Intercept.	Age	−0.1435	−211.4666	62.2053	−3.3995	−0.2479	0.0002
.Time.	Time	−0.0646	−5.7518	1.4236	−4.0404	−0.2886	0.0002
.Time.	Sex Male:Time	−0.0616	−3.0052	1.0318	−2.9126	−0.2080	0.0004

Results of the GLMM and GEE methods are reported in Tables 3 and 4, respectively. In the GLMM case, we consider both intercept and random slopes; the results assuming only a random intercept are placed in Appendix B. In *flip2sss*, the statistical test related to Ethiopia is the significant one, while in GEE, Cambodia and India are the only nonsignificant coefficients. This is also true for the GLMM with the intercept as random effect only (see Appendix B), while by modeling both intercept and slopes as random, the nonsignificant ones are China, Cambodia, and India (see Table 3). This again emphasizes the problem that the choice of the random part’s structure affects the results. This problem is bypassed by adopting the proposed *flip2sss* approach.

**Table 3:** GLMM model results: summary of the model and related analysis of variance table.

	**Estimate**	**Std. Error**	*z* **value**	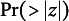
Intercept	3.0496	0.4356	7.000	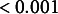
Time	−0.1923	0.0186	−10.317	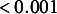
Sex Male	0.8843	0.2545	3.475	0.0005
Country Cambodia	−1.8904	1.0796	−1.751	0.0799
Country China	−0.4613	0.4485	−1.028	0.3038
Country Colombia	−2.9043	0.3897	−7.452	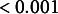
Country Ethiopia	−2.5162	0.4400	−5.718	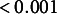
Country India	−0.3485	0.4684	−0.744	0.4568
Country Thailand	−3.1642	0.5867	−5.393	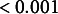
Country Vietnam	−1.5367	0.5765	−2.666	0.0077
Age	−0.1811	0.0357	−5.068	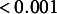
Time: Sex: Male	−0.0298	0.0184	−1.617	0.1058

**Groups**	**Name**	**Variance**	**Std.Dev.**	**Corr**
Subject	(Intercept)	2.6797	1.637	
	Time	0.0068	0.0822	−0.33

	**n. par**	**Sum Sq.**	**Mean Sq.**	*F* **value**
Time	1	287.08	287.08	287.08
Sex	1	10.53	10.53	10.53
Country	7	148.82	21.26	21.26
Age	1	28.38	28.38	28.38
Time: Sex	1	2.34	2.34	2.34

**Table 4:** GEE results: summary of the model and related analysis of variance table.

	**Estimate**	**Std.err**	**Wald**	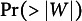
Intercept	2.0834	0.2744	57.67	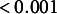
Time	−0.1132	0.0097	137.35	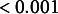
Sex Male	0.5969	0.1562	14.60	0.0001
Country Cambodia	−1.2498	0.7839	2.54	0.1109
Country China	−0.5890	0.2494	5.58	0.0182
Country Colombia	−2.1148	0.2330	82.35	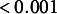
Country Ethiopia	−1.8077	0.2665	46.00	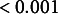
Country India	−0.3305	0.2634	1.57	0.2095
Country Thailand	−2.2608	0.3357	45.36	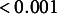
Country Vietnam	−0.9533	0.3392	7.90	0.005
Age	−0.1210	0.0182	44.17	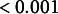
Time: Sex: Male	−0.0236	0.0118	4.02	0.0451

## 6 Discussion

The extension of the *flipscores* (De Santis et al., 2024; Hemerik et al., 2020) method proposed here (i.e., *flip2sss*) accounts for heteroscedasticity between- and within-subject dependence, which are crucial considerations in statistical modeling. With existing methods, the heteroscedastic nature of data can introduce bias and compromise the validity of statistical inferences. Accounting for within-subject dependence is likewise essential for valid statistical inference. The proposed method is shown to have the required properties through simulations and real-world applications. These demonstrate our method’s superiority in terms of statistical power and control over false positive rates compared to conventional parametric methods in several settings. In addition, the proposed approach is able to handle several random effects without an explicit specification in the model. The researcher is not required to choose the random structure of the model (i.e., random intercept and random slope or related quantities), as happens when the GLM or other common “two-stage summary statistics” approaches proposed in the literature (e.g., Beckmann et al., 2003; Finos and Basso, 2014; Frison and Pocock, 1992; Helwig, 2019; Mumford and Poldrack, 2007) are used. The only prerequisite is a grasp of the underlying structure of data clusters in order to properly estimate the summary measures at the subject level in the first stage. In contrast, specifying the random part of a GLMM is a complex process: no general solution is available for the problem of choosing between, e.g., a parsimonious (Bates, Kliegl, et al., 2015; Matuschek et al., 2017) and a maximal (Barr et al., 2013) model. This choice, however, directly impacts the inferential conclusions. This happens in some way also using the GEE approach, since it assumes a working correlation that is barely known in practice, and choosing the identity (i.e., assuming independence between the repeated measurements) is a safe choice to have consistent estimators (Sullivan Pepe & Anderson, 1994).

Some limitations of the approach are to be discussed. The correctness of the method depends directly on the quality estimation of the subject-level summary measures in the first stage. An unbiased (at least asymptotically) estimator is, in fact, necessary to properly control the type I error and to have satisfactory power in the second stage. Nevertheless, this is beyond the scope of the article, and future research will be needed to compare the results with different estimates of the subject-level measure and find the most suitable one in terms of type I error and power. Here, we present two common estimators, the maximum likelihood estimator and its Firth correction (Firth, 1995). In addition, this drawback allows some freedom for the researcher to utilize different approaches in the first stage that are capable of handling, for example, perfect separation problems in the logistic model and/or a low number of repeated measurements. Several works are, in fact, present in the literature (Cordeiro & McCullagh, 1991; Kenne Pagui et al., 2017; Kosmidis et al., 2020; Kosmidis & Firth, 2009, 2021) to correct the 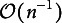 bias where *n* stands for the sample size, i.e., repeated measurements within the cluster in our case. Another limitation includes handling more complex correlation structures such as crossed-random effects (Anderson & Braak, 2003). The cases described above and, in addition, the extension to the multivariate case (i.e., when we have more than one dependent variable) will be properly investigated in further works.

In conclusion, our research extends the applicability of permutation-based approaches in generalized linear mixed models, offering a flexible and reliable tool to overcome the limitations of traditional statistical parametric methods. With its ability to handle complex random effect structures and account for heteroscedasticity and within-subject dependence, our approach promises to advance statistical inference in psychometric research, neuroscience, and beyond. As such, it opens up new avenues for exploring intricate relationships and dependencies within diverse datasets, expanding the horizons of statistical analysis in various fields.

## Figures and Tables

**Figure A1:**
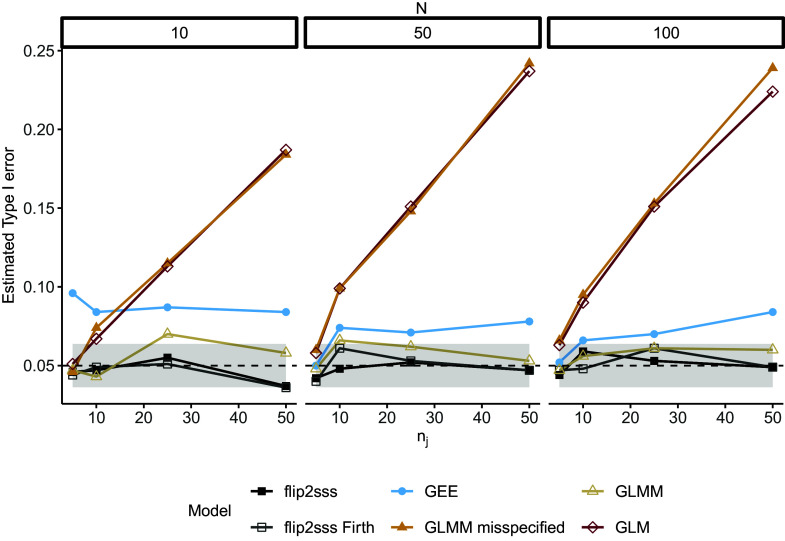
Estimated type I error considering 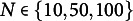 number of subjects and 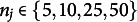 repeated measurements. Each line represents one model, and the grey area around the dashed black line represents the 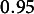 confidence bounds for 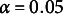 . The term “GLMM” refers to the GLMM with random intercept and random slope specified, whereas “misspecified GLMM” refers to the GLMM with solely the random intercept included in the model specification. The term “flip2sss” refers to the results using the classic GLM in the first stage, while the term “flip2sss Firth” refers to the results using the bias correction proposed by Firth (1995). Finally, the terms GLM and GEE refer to a GLM with a logit link function and a GEE with an independent working correlation matrix, respectively.

**Figure A2:**
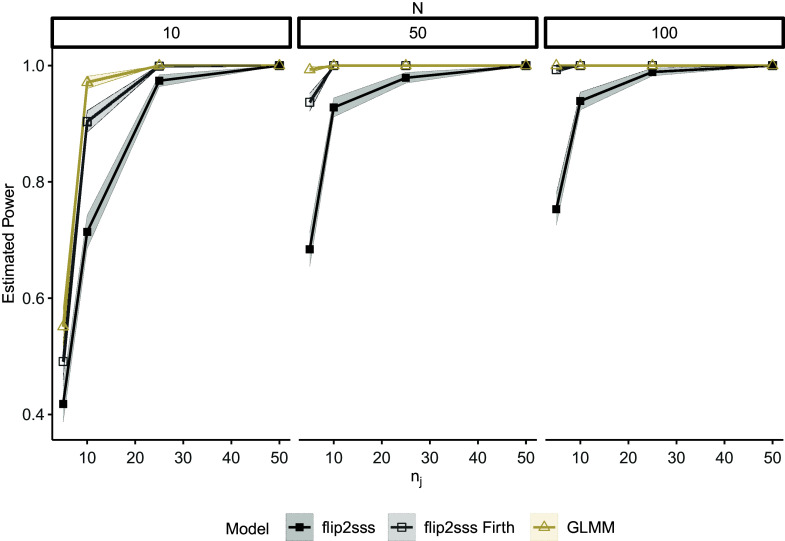
Estimated power considering 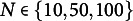 number of subjects and 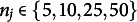 repeated measurements. Each line represents one model with corresponding colored confidence bounds at level 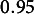 . The term “flip2sss” refers to the results using the classic GLM in the first stage, while the term “flip2sss Firth” refers to the results using the bias correction proposed by Firth (1995).

**Figure A3:**
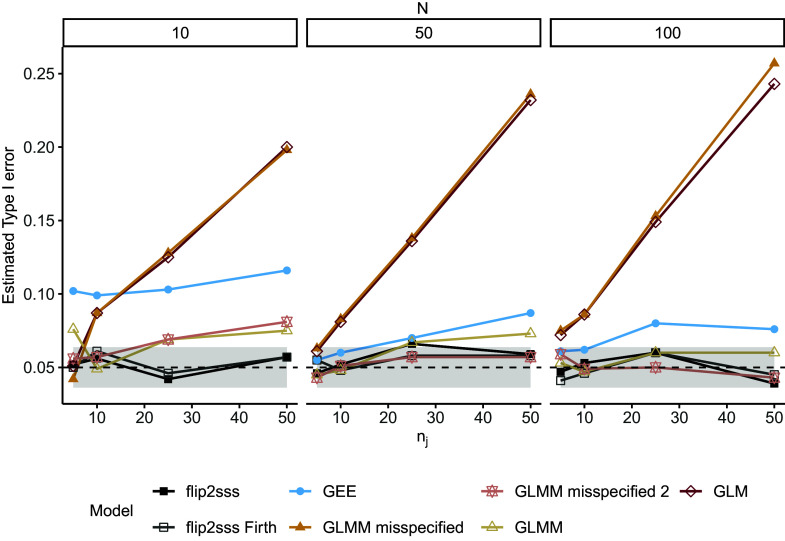
Estimated type I error considering 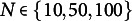 number of subjects and 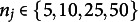 repeated measurements. Each line represents one model, and the grey area around the dashed black line represents the 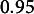 confidence bounds for 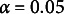 . The term “misspecified GLMM 2” refers to the GLMM with random intercept and random slope for 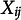 specified, whereas “misspecified GLMM” refers to the GLMM with solely the random intercept included in the model specification. The term “flip2sss” refers to the results using the classic GLM in the first stage, while the term “flip2sss Firth” refers to the results using the bias correction proposed by Firth (1995). Finally, the terms GLM and GEE refer to a GLM with a logit link function and a GEE with an independent working correlation matrix, respectively.

**Figure A4:**
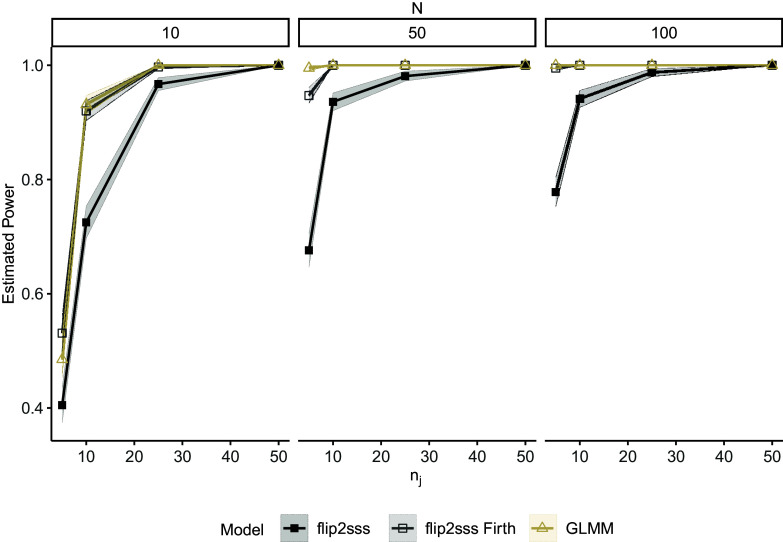
Estimated power considering 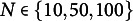 number of subjects and 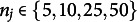 repeated measurements. Each line represents one model with corresponding colored confidence bounds at level 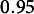 . The term “flip2sss” refers to the results using the classic GLM in the first stage, while the term “flip2sss Firth” refers to the results using the bias correction proposed by Firth (1995).

**Figure A5:**
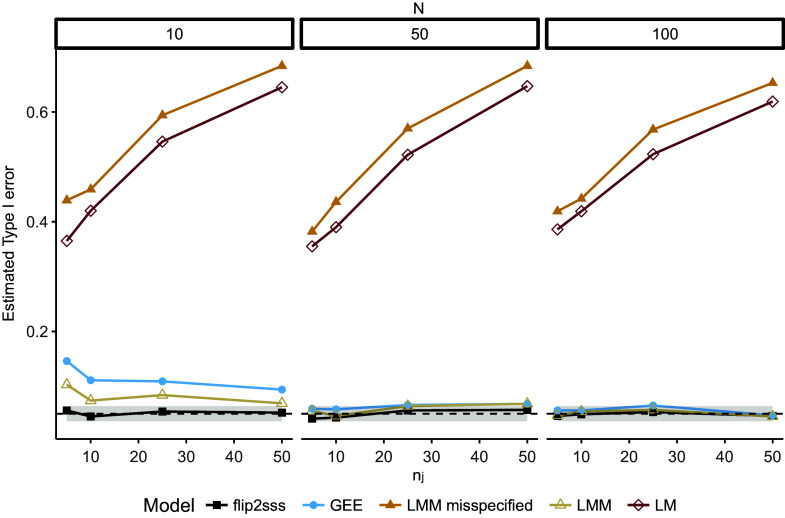
Estimated type I error considering 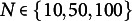 number of subjects and 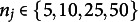 repeated measurements in the case of heteroscedasticity. Each line represents one model, and the grey area around the dashed black line represents the 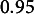 confidence bounds for 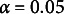 . The “LMM” refers to the LMM specifying random intercept and random slope, while the “LMM misspecified” refers to the LMM with only random intercept. Finally, the terms LM and GEE refer to a normal linear model and a GEE with an independent working correlation matrix, respectively.

**Figure A6:**
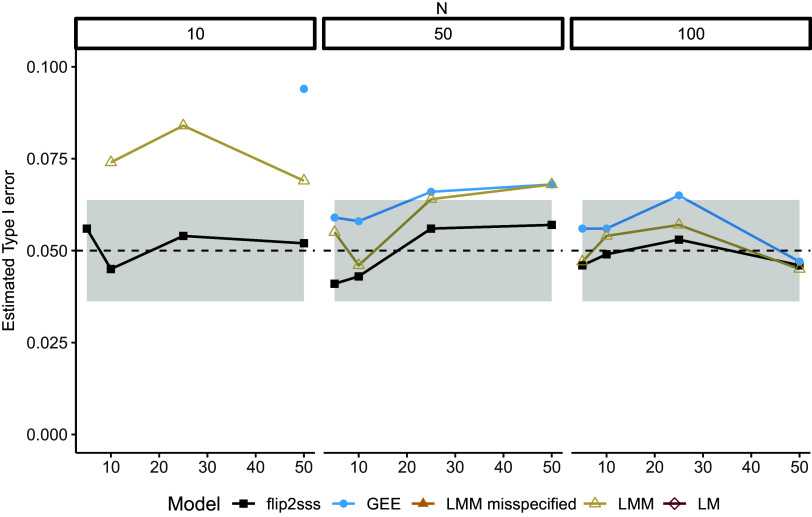
Zoom of Figure A5 considering the estimated type I error 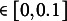 . Estimated type I error considering 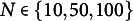 number of subjects and 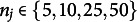 repeated measurements in the case of heteroscedasticity. Each line represents one model, and the grey area around the dashed black line represents the 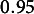 confidence bounds for 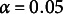 . The “LMM” refers to the LMM specifying random intercept and random slope, while the “LMM misspecified” refers to the LMM with only random intercept. Finally, the terms LM and GEE refer to a normal linear model and a GEE with an independent working correlation matrix, respectively.

## A Simulation results

Here, we present simulations in the case of a subject-specific nuisance covariate. The dependent variable  is then simulated as a Bernoulli random variable with mean:

(A.1)

following the same notations of Equation (7). Here,  and  are simulated from a bivariate normal distribution with mean  and correlation equals  . We have  ,  and  . We perform  simulations for each scenario and, as Section 4, we impose  for estimating the type I error and  for analyzing the power of the approaches, while the value of the nuisance parameter  is fixed at  .

Figure A1 represents the scenario when Equation (A.1) is simulated without the random effect with respect to the within-subject nuisance covariate  , i.e.,  . We can note how GEE does not control the type I error, while the performance of GLMM strongly depends again on the correct specification of the random structure. Here, the “GLMM misspecified” refers to fitting a GLMM without specifying the random slope for  while simulations follow Equation (A.1) with  . The corrected specified GLMM returns  of the times a singularity warning and  of the times a non-convergence issue. In these cases, we consider a *p*-value equals  . Finally, Figure A2 shows the estimated power. Even if the *flip2sss* Firth method loses some power in the case of a small number of repeated measurements, the trend follows roughly the one of the correctly specified GLMM.

Figure A3 shows the estimated type I error simulating  as Equation (A.1), i.e., with random slope for both  and  . In this case, three GLMMs are fitted: (1) considering only random intercept named “GLMM misspecified”, (2) considering random intercept and random slope for  (i.e., the random slope for  is not specified, named “GLMM misspecified 2”) and (3) considering random intercept and the two random slopes for  and  (i.e., the correctly specified GLMM, named simply “GLMM”) in the model. We can note that GEE fails to control the type I error in any scenario, while again, the performance of GLMM depends on the specification of the random structure of the model. In contrast, the two approaches proposed, i.e., *flip2sss* with or without the Firth correction, control properly the type I error, maintaining a good power as shown in Figure A4.

Figure A5 shows the estimated type I error following the settings of Figure 1 but assuming  with  and  . Figure A6 focuses on estimated error values ranging from  to  , providing a clearer comparison of the methods’ ability to control type I error. We can note how the *flip2sss* approach proposed is able to deal with heteroscedasticity and small sample size at the same time in contrast to GEE and Linear Mixed Model (LMM). Here, the lmerTest package (Kuznetsova, 2017) is used to compute tests on lmer objects.

## B Application

Table B1 represents the same results as Table 3 assuming only the random intercept instead of random intercept plus random slope (i.e., the modGLMM2 object defined in Code 2).

**Table B1:** GLMM results assuming only the random intercept: summary of the model and related analysis of variance table.

	**Estimate**	**Std. Error**	*z* **value**	
Intercept	2.7712	0.4024	6.89	
Time	−0.1501	0.0122	−12.26	
Sex Male	0.8847	0.2419	3.66	0.0003
Country Cambodia	−1.7386	1.0144	−1.71	0.0865
Country China	−0.8148	0.3964	−2.06	0.0398
Country Colombia	−2.9041	0.3552	−8.18	
Country Ethiopia	−2.5162	0.4117	−6.11	
Country India	−0.5482	0.4219	−1.30	0.1938
Country Thailand	−3.1516	0.5585	−5.64	
Country Vietnam	−1.3671	0.5258	−2.60	0.0093
Age	−0.1638	0.0339	−4.84	
Time: Sex: Male	−0.0328	0.0150	−2.19	0.0287

**Groups**	**Name**	**Variance**	**Std. Dev.**
Subject	(Intercept)	2.288	1.5126

	**n. par**	**Sum Sq.**	**Mean Sq.**	*F* **value**
Time	1	277.43	277.43	277.43
Sex	1	8.50	8.50	8.50
Country	7	143.46	20.49	20.49
Age	1	25.84	25.84	25.84
Time: Sex	1	4.54	4.54	4.54

In Code 2, we report the R commands used to estimate the GLMMs and GEE.

## References

[r1] Agresti, A. (2015). Foundations of linear and generalized linear models. John Wiley & Sons.

[r2] Albert, A. , & Anderson, J. A. (1984). On the existence of maximum likelihood estimates in logistic regression models. Biometrika, 71(1), 1–10.

[r3] Anderson, M. , & Braak, C. T. (2003). Permutation tests for multi-factorial analysis of variance. Journal of Statistical Computation and Simulation, 73(2), 85–113.

[r4] Azzalini, A. (2017). Statistical inference based on the likelihood. Routledge.

[r5] Barr, D. J. , Levy, R. , Scheepers, C. , & Tily, H. J. (2013). Random effects structure for confirmatory hypothesis testing: Keep it maximal. Journal of Memory and Language, 68(3), 255–278. 10.1016/j.jml.2012.11.001 PMC3881361 24403724

[r6] Basso, D. , & Finos, L. (2012). Exact multivariate permutation tests for fixed effects in mixed-models. Communications in Statistics-Theory and Methods, 41(16-17), 2991–3001.

[r7] Bates, D. , Kliegl, R. , Vasishth, S. , & Baayen, H. (2015). Parsimonious mixed models. arXiv preprint arXiv:1506.04967.

[r8] Bates, D. , Mächler, M. , Bolker, B. , & Walker, S. (2015). Fitting linear mixed-effects models using lme4. Journal of Statistical Software, 67, 1–48.

[r9] Beckmann, C. F. , Jenkinson, M. , & Smith, S. M. (2003). General multilevel linear modeling for group analysis in FMRI. Neuroimage, 20(2), 1052–1063. 14568475 10.1016/S1053-8119(03)00435-X

[r10] Bryson, M. C. , & Johnson, M. E. (1981). The incidence of monotone likelihood in the cox model. Technometrics, 23(4), 381–383.

[r11] Carlin, J. B. , Wolfe, R. , Coffey, C. , & Patton, G. C. (1999). Analysis of binary outcomes in longitudinal studies using weighted estimating equations and discrete-time survival methods: prevalence and incidence of smoking in an adolescent cohort. Statistics in Medicine, 18(19), 2655–2679. 10495463 10.1002/(sici)1097-0258(19991015)18:19<2655::aid-sim202>3.0.co;2-#

[r12] Cnaan, A. , Laird, N. M. , & Slasor, P. (1997). Using the general linear mixed model to analyse unbalanced repeated measures and longitudinal data. Statistics in Medicine, 16(20), 2349–2380. 9351170 10.1002/(sici)1097-0258(19971030)16:20<2349::aid-sim667>3.0.co;2-e

[r13] Cordeiro, G. M. , & McCullagh, P. (1991). Bias correction in generalized linear models. Journal of the Royal Statistical Society Series B: Statistical Methodology, 53(3), 629–643.

[r14] Crowder, M. (1995). On the use of a working correlation matrix in using generalised linear models for repeated measures. Biometrika, 82(2), 407–410.

[r15] De Santis, R. , Goeman, J. J. , Hemerik, J. , & Finos, L. (2024). Inference in generalized linear models with robustness to misspecified variances. arXiv preprint arXiv:2209.13918. 10.1080/01621459.2025.2491775 PMC12758902 41488331

[r16] Diggle, P. , Liang, K.-Y. , & Zeger, S. L. (1994). Longitudinal data analysis. New York: Oxford University Press, 5, 13.

[r17] Everitt, B. (1995). The analysis of repeated measures: a practical review with examples. Journal of the Royal Statistical Society Series D: The Statistician, 44(1), 113–135.

[r18] Finos, L. , & Andreella, A. (2024). Jointest: Multivariate testing through joint resampling-based tests [Computer software manual]. Retrieved from https://CRAN.R-project.org/package=jointest (R package version 1.0)

[r19] Finos, L. , & Basso, D. (2014). Permutation tests for between-unit fixed effects in multivariate generalized linear mixed models. Statistics and Computing, 24, 941–952.

[r20] Firth, D. (1995). Bias reduction of maximum likelihood estimates. Biometrika, 82(3), 667–667.

[r21] Fox, J. , & Weisberg, S. (2019). An R companion to applied regression. (3rd ed.). Sage.

[r22] Frison, L. , & Pocock, S. J. (1992). Repeated measures in clinical trials: analysis using mean summary statistics and its implications for design. Statistics in Medicine, 11(13), 1685–1704. 1485053 10.1002/sim.4780111304

[r23] Gardiner, J. C. , Luo, Z. , & Roman, L. A. (2009). Fixed effects, random effects and gee: What are the differences? Statistics in Medicine, 28(2), 221–239. 19012297 10.1002/sim.3478

[r24] Heagerty, P. J. , & Kurland, B. F. (2001). Misspecified maximum likelihood estimates and generalised linear mixed models. Biometrika, 88(4), 973–985.

[r25] Heinze, G. , & Schemper, M. (2002). A solution to the problem of separation in logistic regression. Statistics in Medicine, 21(16), 2409–2419. 12210625 10.1002/sim.1047

[r26] Helwig, N. E. (2019). Statistical nonparametric mapping: Multivariate permutation tests for location, correlation, and regression problems in neuroimaging. Wiley Interdisciplinary Reviews: Computational Statistics, 11(2), e1457.

[r27] Hemerik, J. , Goeman, J. J. , & Finos, L. (2020). Robust testing in generalized linear models by sign flipping score contributions. Journal of the Royal Statistical Society Series B: Statistical Methodology, 82(3), 841–864.

[r28] Højsgaard, S. , Halekoh, U. , & Yan, J. (2006). The r package geepack for generalized estimating equations. Journal of statistical software, 15, 1–11.

[r29] Kenne Pagui, E. C. , Salvan, A. , & Sartori, N. (2017). Median bias reduction of maximum likelihood estimates. Biometrika, 104(4), 923–938.

[r30] Kherad-Pajouh, S. , & Renaud, O. (2010). An exact permutation method for testing any effect in balanced and unbalanced fixed effect anova. Computational Statistics & Data Analysis, 54(7), 1881–1893.

[r31] Kosmidis, I. (2023). brglm2: Bias reduction in generalized linear models [Computer software manual]. Retrieved from https://CRAN.R-project.org/package=brglm2 (R package version 0.9.2)

[r32] Kosmidis, I. , & Firth, D. (2009). Bias reduction in exponential family nonlinear models. Biometrika, 96(4), 793–804.

[r33] Kosmidis, I. , & Firth, D. (2021). Jeffreys-prior penalty, finiteness and shrinkage in binomial-response generalized linear models. Biometrika, 108(1), 71–82.

[r34] Kosmidis, I. , Kenne Pagui, E. C. , & Sartori, N. (2020). Mean and median bias reduction in generalized linear models. Statistics and Computing, 30(1), 43–59.

[r35] Kuznetsova, A. , Brockhoff, P. B. , & Christensen, R. H. B. (2017). lmerTest package: Tests in linear mixed effects models. Journal of Statistical Software, 82(13), 1–26. 10.18637/jss.v082.i13.

[r36] Lagarias, J. C. , Reeds, J. A. , Wright, M. H. , & Wright, P. E. (1998). Convergence properties of the nelder–mead simplex method in low dimensions. SIAM Journal on Optimization, 9(1), 112–147.

[r37] Laird, N. M. , & Ware, J. H. (1982). Random-effects models for longitudinal data. Biometrics, 38(4), 963–974. 7168798

[r38] Lee, O. E. , & Braun, T. M. (2012). Permutation tests for random effects in linear mixed models. Biometrics, 68(2), 486–493. 21950470 10.1111/j.1541-0420.2011.01675.x PMC3883440

[r39] Liang, K.-Y. , & Zeger, S. L. (1986). Longitudinal data analysis using generalized linear models. Biometrika, 73(1), 13–22.

[r40] Matuschek, H. , Kliegl, R. , Vasishth, S. , Baayen, H. , & Bates, D. (2017). Balancing type i error and power in linear mixed models. Journal of Memory and Language, 94, 305–315.

[r41] Mumford, J. A. , & Poldrack, R. A. (2007). Modeling group fmri data. Social Cognitive and Affective Neuroscience, 2(3), 251–257. 18985145 10.1093/scan/nsm019 PMC2569805

[r42] Pesarin, F. (2001). Multivariate permutation tests: with applications in biostatistics. Chichester: Wiley, 240.

[r43] Powell, M. J. , et al. (2009). The bobyqa algorithm for bound constrained optimization without derivatives. University of Cambridge, 26. NA Report NA2009/06.

[r44] Puhr, R. , Heinze, G. , Nold, M. , Lusa, L. , & Geroldinger, A. (2017). Firth’s logistic regression with rare events: accurate effect estimates and predictions? Statistics in Medicine, 36(14), 2302–2317. 28295456 10.1002/sim.7273

[r45] Santona, A. , Tognasso, G. , Miscioscia, C. L. , Russo, D. , & Gorla, L. (2022). Talking about the birth family since the beginning: The communicative openness in the new adoptive family. International Journal of Environmental Research and Public Health, 19(3), 1203. 35162222 10.3390/ijerph19031203 PMC8834835

[r46] Sullivan Pepe, M. , & Anderson, G. L. (1994). A cautionary note on inference for marginal regression models with longitudinal data and general correlated response data. Communications in Statistics-Simulation and Computation, 23(4), 939–951.

[r47] Sutradhar, B. C. , & Das, K. (2000). On the accuracy of efficiency of estimating equation approach. Biometrics, 56(2), 622–625. 10877326 10.1111/j.0006-341x.2000.00622.x

[r48] Tuerlinckx, F. , Rijmen, F. , Verbeke, G. , & De Boeck, P. (2006). Statistical inference in generalized linear mixed models: A review. British Journal of Mathematical and Statistical Psychology, 59(2), 225–255. 17067411 10.1348/000711005X79857

[r49] Vonesh, E. , & Chinchilli, V. M. (1996). Linear and nonlinear models for the analysis of repeated measurements. CRC Press.

[r50] Wang, Y.-G. , & Carey, V. (2003). Working correlation structure misspecification, estimation and covariate design: implications for generalised estimating equations performance. Biometrika, 90(1), 29–41.

